# A Case of Angiotensin-Converting Enzyme (ACE) Inhibitor-Induced Small Bowel Angioedema

**DOI:** 10.7759/cureus.47739

**Published:** 2023-10-26

**Authors:** Sarmad Pirzada, Beebarg Raza, Ali Abbas Mankani, Bazigh Naveed

**Affiliations:** 1 Internal Medicine, Trinity Health Livonia, Livonia, USA; 2 Internal Medicine, Dow University of Health Sciences, Civil Hospital Karachi, Karachi, PAK; 3 Internal Medicine, King Edward Medical University (KEMU), Lahore, PAK

**Keywords:** ace-inhibitor-induced angioedema, medication side effects, side effects, angiotensin-converting enzyme inhibitor, intestinal angioedema, small bowel angioedema

## Abstract

Angioedema is a rare but known side effect of angiotensin-converting enzyme (ACE) inhibitor therapy. The most common presentations of ACE inhibitor-induced angioedema describe swellings in the oropharyngeal and periorbital regions. We describe a rare case of a 58-year-old female with a history of type 2 diabetes and hypertension taking lisinopril for the past three years and presented with recurrent episodes of abdominal pain, nausea, and vomiting around the same time she started taking the drug. Multiple computed tomography (CT) scans were performed, which showed findings consistent with edema in the proximal small bowel. Due to the recurrent nature of these episodes over the last three years, along with consistent findings of small bowel edema on imaging, lisinopril-induced angioedema was suspected. As a result, the patient was switched from lisinopril to amlodipine. During our follow-up with the patient, she reported that her symptoms had resolved following the withdrawal of lisinopril.

## Introduction

Angiotensin-converting enzyme (ACE) inhibitors, such as lisinopril, are one of the most commonly prescribed classes of drugs in the world of medicine. They are used to treat hypertension, heart failure, stroke, chronic kidney disease, and a number of other cardiovascular problems. Side effects of these drugs include dry cough, hyperkalemia, hypotension, headaches, and renal insufficiency secondary to hypotension. Rarely, ACE inhibitors may also cause subcutaneous and submucosal tissues of your body to swell up, a condition known as angioedema. Angioedema is a rare adverse effect of ACE inhibitors that presents in only 0.7% of patients in the first five years of use and only 0.07% of patients within the first month [1,2]. ACE inhibitor-induced angioedema most commonly affects the face, tongue, lips, and upper airways [3]. Isolated small bowel angioedema is an extremely rare complication of ACE inhibitor therapy that often goes clinically undiagnosed, as it commonly presents, both symptomatically and radiographically, similarly to several other illnesses such as small bowel ischemia, enteritis, lymphoma, vasculitis, C1 esterase deficiency, and Crohn’s disease [4]. Symptoms include nausea, vomiting, abdominal pain, and diarrhea [5]. Radiological findings of ACE-inhibitor-induced small bowel angioedema are also non-specific and include dilatation and thickening of the small bowel wall, which are concerning for enteritis [6]. The clinical and radiological findings of ACE-inhibitor-induced angioedema are of extreme significance to recognize the cause of the angioedema and thus avoid unessential investigations and medical interventions. In this case report, we describe a case of a 58-year-old female with small bowel angioedema after three years of lisinopril therapy.

## Case presentation

A 58-year-old female with a history of type 2 diabetes, hypertension, and gastroesophageal reflux disease (GERD) presented to the emergency department with abdominal pain, nausea, and vomiting for one day. She mentioned that she had been experiencing similar recurrent episodes over the last three years with repetitive self-limiting presentations. She denied any change in her bowel habits or any other complaints. The patient did not have any drug or food allergies. She was referred to GI but by the time she was evaluated, she was again asymptomatic and denied any nausea, vomiting, or abdominal pain. Her drug history showed that she had been taking 20 mg of lisinopril every day for the last three years approximately around the same time these episodes began (Table 1).

**Table 1:** The patient’s drug history and the common GI side effects of these drugs

Drugs	Gastrointestinal Side Effects
Chlorthalidone	Abdominal pain and black, tarry stools
Lisinopril	Diarrhea, nausea, vomiting, small bowel angioedema (rare)
Glimepiride	Abdominal pain
Metformin	Diarrhea, bloating, abdominal pain, and constipation
Oxybutynin	Diarrhea and nausea
Sertraline	Diarrhea and nausea
Trazodone	Nausea and vomiting

On admission, the patient was afebrile and her vital signs were normal. Routine laboratory workup, including a complete blood count, complete metabolic profile, and pancreatic enzymes, showed that the patient had low hemoglobin (10.5 g/dL), low hematocrit (33%), low total bilirubin (0.2 mg/dL), low glomerular filtration rate (GFR) (62.0 ml/min/1.73 m^2), and elevated blood glucose (163 mg/dL). All other routine laboratory studies were within normal limits (Table 2). The patient’s tissue transglutaminase immunoglobulin A antibody (tTG-IgA) test, clostridium difficile toxin, fecal leukocytes, and calprotectin were also negative.

**Table 2:** Initial laboratory results upon presentation BUN: blood urea nitrogen; GFR: glomerular filtration rate; AST: aspartate aminotransferase; ALT: alanine transaminase; MCV: mean corpuscular volume; MCHC: mean corpuscular hemoglobin concentration; RDW: red blood cell distribution width

Lab	Results	Reference
Albumin	3.7 mg/dL	3.5-5.5 mg/dL
Total Protein	7.2 g/dL	6.0-8.5 g/dL
BUN	13.0 mg/dL	6.0-24.0 mg/dL
Creatinine	1.05 mg/dL	0.6-1.1 mg/dL
BUN/Creatinine Ratio	12.4	10.0-20.0
GFR	62.0 ml/min/1.73 m^2	90.0-120.0 ml/min/1.73 m^2
AST	26.0 IU/L	5.0-40.0 IU/L
ALT	33.0 IU/L	10.0-40.0 IU/L
Alkaline Phosphatase	59.0 IU/L	0-130 IU/L
Hemoglobin	10.5 g/dL	11.1-14.5 g/dL
Hematocrit	33.0%	35.4-42.0%
MCV	81.3	76.0-96.0 fL
RBC	4.06 ×10^6/microL	3.9-5.5 ×10^6/microL
MCHC	31.8 g/dL	32.0-36.0 g/dL
RDW	13.0	12.0-15.0%
Platelets	242	150-400 × 10^9/L
WBC	6.6 × 10^9/L	4.0-10.0 × 10^9/L
Total Bilirubin	0.2 mg/dL	0.0-1.2 mg/dL
Sodium	137.0 mmol/L	136-145 mmol/L
Potassium	3.6 mmol/L	3.5-5.0 mmol/L
Magnesium	1.8 mg/dL	1.7 to 2.2 mg/dL
Chloride	104.0 mmol/L	95-106 mmol/L
CO2	22.0 mEq/L	23.0-29.0 mEq/L
Anion Gap	11.0 mmol/L	4.0-12.0 mmol/L
Glucose	163.0 mg/dL	65-99.0 mg/dL

**Figure 1:**
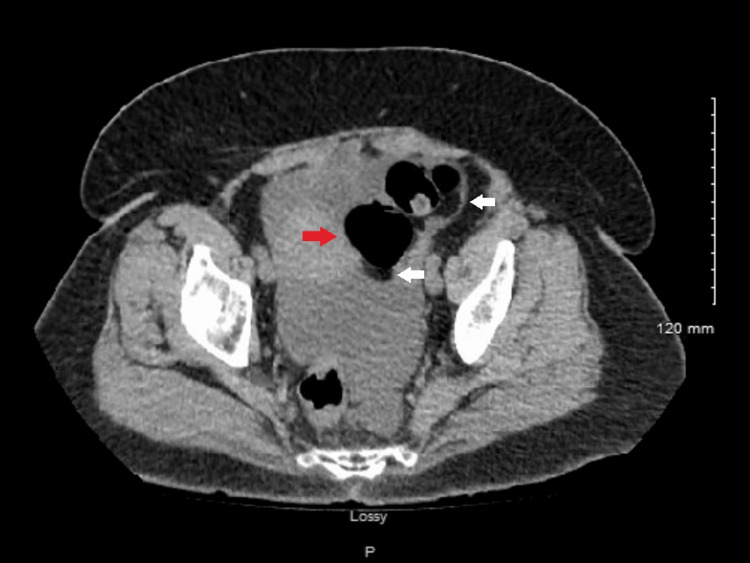
CT angio abdomen and pelvis (axial view) White arrows demonstrate wall thickening with edema along the proximal and mid small bowel. The red arrow demonstrates increased enhancement along the small bowel loop.

Multiple computerized axial tomography (CAT) scans were performed over the last three years but interestingly, only the most recent ones showed similar findings of edema of the proximal small bowel. The most recent contrast-enhanced CT scan of the abdomen and pelvis revealed thickening of the small bowel wall with edema along the proximal and mid small bowel along with adjacent fluid and a small amount of ascites. No pneumatosis intestinalis, discrete abscess collection, free intraperitoneal gas, or portal venous gas was identified (Figure 1). The findings of the CT scan were most suggestive of infectious vs inflammatory enteritis. Findings are similar to previous CT scans of the abdomen performed at prior admissions.

Due to the recurrent nature of the patient’s episodes and multiple admissions over the last three years, lisinopril-induced small bowel angioedema was suspected. As part of our management strategy, lisinopril was discontinued and the patient was switched to amlodipine. Another CT scan of the abdomen and pelvis was performed the next day, which revealed substantial improvement in the proximal small bowel enteritis in comparison to the day before. This finding was highly favored to reflect ACE-inhibitor-induced small bowel angioedema. As a result, the patient was recommended to cease her ACE inhibitor therapy and switch to amlodipine. During follow-up, the patient reported that her symptoms had remained stable since discontinuing lisinopril.

## Discussion

Millions of patients around the world are prescribed ACE inhibitors for various illnesses every day. While the risk of angioedema secondary to ACE inhibitor therapy is very low, it is one of the most well-known side effects of this class of drugs. This is perhaps because ACE inhibitor therapy is the leading cause of drug-induced angioedema in the United States [7].

With millions of patients in the US being treated with anti-hypertensive drugs, at least one in three are prescribed an ACE inhibitor such as lisinopril during their lifetime [8]. In addition, ACE inhibitors are prescribed to manage a number of other illnesses such as diabetes mellitus, congestive heart failure, and chronic kidney disease. With such a significant number of patients taking ACE inhibitors, one can only expect thousands of cases of drug-induced angioedema despite the low incidence of this adverse effect. Unfortunately, this side effect of ACE inhibitor therapy is severely underreported and only very few cases have been reported in literature.

ACE-inhibitor-induced angioedema usually involves the tongue, lips, face, and upper airways. Isolated small bowel involvement is an extremely rare presentation of this side effect with very few cases that have been reported in previous literature. A review by Palmquist and Mathews found only 34 cases of ACE-inhibitor-induced visceral angioedema from 1980 to 1 August 2016 [9]. Of these cases, 85.3% were female, with an average age of 49.5 years. Lisinopril was also the most commonly reported cause among all the ACE inhibitors.

Angioedema may develop as early as a day after starting ACE inhibitor therapy or after several years of use and is not related to the dose of ACE inhibitor taken. Several mechanisms of action have been proposed when it comes to ACE-inhibitor-induced angioedema. However, the most widely accepted mechanism of action relates to the levels of bradykinin in the body. The angiotensin-converting enzyme is structurally similar to the kininase II enzyme, an enzyme responsible for the degradation of bradykinin into inactive peptides in our body. Pharmacological inhibition of the angiotensin-converting enzyme also causes inhibition of the kininase II enzyme. This prevents the breakdown of bradykinin and thus leads to increased levels of the vasodilator in the body. High levels of bradykinin cause vasodilation and enhance the permeability of postcapillary venules, allowing plasma to extravasate into the submucosal tissue, resulting in angioedema [3,10]. Patients with hereditary angioedema due to a C1 esterase inhibitor deficiency are also known to have high levels of bradykinin [11]. Hereditary angioedema presents with low levels of C4 and C1 esterase inhibitors [12]. Thus, in cases presenting with small bowel angioedema alone, it is extremely important to rule out C1 esterase inhibitor deficiency by obtaining C4 and C1 inhibitor antigen levels and functional essays for C1 esterase inhibitor activity [13].

Symptoms of ACE-inhibitor-induced angioedema are usually non-specific and self-limiting. These include episodes of abdominal pain, nausea, vomiting, and loose stools. Rarely, the patient may experience diarrhea and ascites [5]. Unlike other drug reactions, these bouts of symptoms due to ACE-inhibitor-induced small bowel angioedema are inconsistent and resolve within two to three days even without discontinuation of ACE inhibitor therapy. This is perhaps why ACE-inhibitor-induced isolated small bowel angioedema is often misdiagnosed and patients continue to take their prescribed ACE inhibitor even after multiple episodes of angioedema. Continuing with ACE inhibitor therapy after the first bout of angioedema can severely increase the risk of morbidity [14]. In our case, the patient continued taking lisinopril for three years before her illness was suspected as a case of ACE-inhibitor-induced small bowel angioedema. During her previous workups, there were no objective signs or radiographic evidence of small bowel angioedema prior to her diagnosis.

Other than a very mild elevation in white blood cell count, laboratory results in such cases are usually normal, as they were in our case. A diagnosis is usually made with the help of the patient’s medication history paired with CT imaging, which confirms edema of the duodenum, jejunum, and ileum with or without ascites. CT findings may show circumferential small bowel thickening, straightening of the bowel loops, and mesenteric edema [4]. The list of differentials in a patient presenting with acute diffuse abdominal pain, nausea, and vomiting is very long. This includes colitis, gastroenteritis, trauma, hypoalbuminemia, Crohn’s disease, vasculitis, lymphoma, ischemia, and C1 esterase deficiency [4].

Although rare and underreported, a few cases of lisinopril-induced small bowel angioedema have been previously reported in the literature. Johnson et al. reported a similar case of lisinopril-induced small bowel angioedema in a 63-year-old female who had been taking lisinopril for seven years to treat her hypertension prior to presentation and had previously consulted physicians for abdominal pain [15]. Her symptoms improved with cessation of lisinopril although she did experience abdominal pain only once shortly after her treatment regimen was changed.

Mujer et al. described a case of a 42-year-old man who presented with acute left lower quadrant abdominal pain, nausea, intractable vomiting, dizziness, and dyspnea [16]. Empirical therapy was initiated with ciprofloxacin and metronidazole. However, the patient's symptoms did not resolve. A careful review of the patient’s history showed that he was recently prescribed lisinopril two weeks prior to the onset of his symptoms. His symptoms improved within 24 hours of discontinuation of lisinopril. 

Squillante et al. reported a case of a 40-year-old Caucasian female who presented to the ED with acute abdominal pain, emesis, nausea, bloating, and moderate generalized abdominal tenderness [1]. Her daily medications included lisinopril 20 mg daily, which she had begun taking three days before the onset of her symptoms, and an oral contraceptive. Her CBC showed a mild leukocytosis of 13,000 while the rest of her labs were normal. CT findings revealed diffuse wall thickening, hyperenhancement, and mucosal edema of the entire small bowel. Lisinopril was discontinued immediately, and the patient’s symptoms gradually resolved.

Another case report described a similar case of a 61-year-old female with a history of asthma and hypertension who presented to the ED with severe episodic abdominal pain. Unlike our case, in which the patient had been experiencing her symptoms for the last three years, this patient presented with a history of pain for the last two months [17]. The patient’s drug history prior to admission included lisinopril 20 mg daily. Following a CT scan and a consult with the radiologist, lisinopril-induced angioedema of the small bowel was suspected. Thus, lisinopril was discontinued, the patient was made NPO with bowel rest, and the patient’s symptoms resolved spontaneously the next day.

Wilin et al. discussed a case of a 62-year-old African American female with a two-year history of lisinopril use who presented with nausea and intermittent left middle and upper quadrant abdominal pain [18]. Her pain worsened with food intake and improved with bowel movements, a feature not present in our case. Similar to our case, the CT scan of the patient’s abdomen showed segmental small bowel thickening and edema with associated ascites and no obstruction, ileus, or lymphadenopathy. Lisinopril therapy was withdrawn and metoprolol tartrate was replaced by carvedilol 12.5 mg orally twice daily. The patient’s symptoms improved within two days without any other supportive treatment.

Another case describes an incident of visceral angioedema in a patient within 48 hours of starting lisinopril therapy [19]. The 41-year-old Caucasian female presented with intermittent repeated bouts of severe lower quadrant abdominal pain, nausea, vomiting, and watery diarrhea. Of note is that our patient reported no change in bowel movements, unlike this case. CT scan of the abdomen and pelvis revealed small bowel edema, a prominent adrenal gland, and ascites. Lisinopril was discontinued and within 48 hours, her symptoms were resolved. Upon follow-up two months later, her CT scans were negative and showed no signs of ascites or bowel edema.

A very peculiar case by Mir and Sorrentino reported a young female with a history of hypertension who presented with left lower quadrant abdominal pain [20]. The 23-year-old patient’s CT scan showed extensive and homogenous jejunal wall thickening. Her drug history showed that she had been put on lisinopril therapy a few weeks prior to the presentation of her symptoms. Lisinopril therapy was discontinued following suspicion of lisinopril-induced angioedema. Follow-up at one month revealed an improvement of the young patient’s symptoms entirely and MRI imaging showed complete resolution of the previous picture.

## Conclusions

Isolated small bowel angioedema is a rare, yet important, adverse reaction of ACE inhibitor therapy. It is imperative to recognize this side effect considering that it is one of the most commonly prescribed drugs in the US and because of the potential complications of this side effect. Since the symptoms of ACE-inhibitor-induced angioedema are similar to several other illnesses, it may lead to a misdiagnosis in many cases resulting in unnecessary procedures and workups. Knowledge of this side effect, a detailed drug history, and CT scan findings can help a physician make the correct diagnosis and resolve the patient’s symptoms by discontinuing the ACE inhibitor with an alternative class of drug such as a calcium channel blocker for their treatment. In this report, we described a rare case of ACE-inhibitor-induced small bowel angioedema where the patient had been on lisinopril for three years before a diagnosis was made and the patient had multiple bouts of abdominal pain and nausea without any evidence of angioedema prior to diagnosis. We hope that this case report adds value to the current literature on ACE-inhibitor-induced side effects.
